# Physiological, genomic and transcriptional diversity in responses to boron deficiency in rapeseed genotypes

**DOI:** 10.1093/jxb/erw342

**Published:** 2016-09-17

**Authors:** Yingpeng Hua, Ting Zhou, Guangda Ding, Qingyong Yang, Lei Shi, Fangsen Xu

**Affiliations:** ^1^National Key Laboratory of Crop Genetic Improvement, Huazhong Agricultural University, Wuhan 430070, China; ^2^Microelement Research Centre, Huazhong Agricultural University, Wuhan 430070, China; ^3^College of Informatics, Huazhong Agricultural University, Wuhan 430070, China

**Keywords:** B-deficiency phenotype, boron (B) efficiency, *Brassica napus*, differentially expressed genes, genomic variations, next-generation sequencing.

## Abstract

Allotetraploid rapeseed (*Brassica napus* L. A_n_A_n_C_n_C_n_, 2*n*=4*x*=38) is highly susceptible to boron (B) deficiency, a widespread limiting factor that causes severe losses in seed yield. The genetic variation in the sensitivity to B deficiency found in rapeseed genotypes emphasizes the complex response architecture. In this research, a B-inefficient genotype, ‘Westar 10’ (‘W10’), responded to B deficiencies during vegetative and reproductive development with an over-accumulation of reactive oxygen species, severe lipid peroxidation, evident plasmolysis, abnormal floral organogenesis, and widespread sterility compared to a B-efficient genotype, ‘Qingyou 10’ (‘QY10’). Whole-genome re-sequencing (WGS) of ‘QY10’ and ‘W10’ revealed a total of 1 605 747 single nucleotide polymorphisms and 218 755 insertions/deletions unevenly distributed across the allotetraploid rapeseed genome (~1130Mb). Digital gene expression (DGE) profiling identified more genes related to B transporters, antioxidant enzymes, and the maintenance of cell walls and membranes with higher transcript levels in the roots of ‘QY10’ than in ‘W10’ under B deficiency. Furthermore, based on WGS and bulked segregant analysis of the doubled haploid (DH) line pools derived from ‘QY10’ and ‘W10’, two significant quantitative trait loci (QTLs) for B efficiency were characterized on chromosome C2, and DGE-assisted QTL-seq analyses then identified a nodulin 26-like intrinsic protein gene and an ATP-binding cassette (ABC) transporter gene as the corresponding candidates regulating B efficiency. This research facilitates a more comprehensive understanding of the differential physiological and transcriptional responses to B deficiency and abundant genetic diversity in rapeseed genotypes, and the DGE-assisted QTL-seq analyses provide novel insights regarding the rapid dissection of quantitative trait genes in plant species with complex genomes.

## Highlight

A comprehensive examination is made of physiological and transcriptional variations and genetic diversity of rapeseed genotypes differing in their response to boron deficiency, and transcriptomics-assisted QTL-seq analyses are found to expedite the identification of quantitative trait genes in plant species with complex genomes.

## Introduction

Boron (B) is an essential micronutrient for the normal growth and development of higher plants (Warington, 1923). In vascular plants, B is principally involved in the formation and structural integrity of cell walls through cross-linking pectic polysaccharide rhamnogalacturonan II (RG-II) (O’Neill *et al.*, 2001; Bolaños *et al.*, 2004). B deficiency primarily affects developing tissues, with symptoms including the inhibition of root elongation and leaf expansion, and reduced fertility (Shorrocks, 1997; Lordkaew *et al.*, 2011). Soils with low B availability are prevalent worldwide and severe crop failures caused by B deficiencies have occurred in more than 80 countries, involving over 132 crop species (Shorrocks, 1997; Goldbach *et al.*, 2001).

Allotetraploid rapeseed (*Brassica napus* L., A_n_A_n_C_n_C_n_, ~1130Mb, 2*n*=4*x*=38) originated from spontaneous interspecific hybridization between the diploid progenitors *B. rapa* (A_r_A_r_, ~485Mb, 2*n*=2*x*=20) and *B. oleracea* (C_o_C_o_, ~630Mb, 2*n*=2*x*=18) about 7500 years ago, followed by chromosome doubling, a process known as allopolyploidy (Chalhoub *et al.*, 2014). These polyploidy events resulted in numerous duplicated segments and homoeologous regions within the *B. napus* genome (Chalhoub *et al.*, 2014), which now cause enormous challenges in the localization of quantitative trait loci (QTLs) for agronomic traits; for example, inaccuracies are caused by homologous sequences from different chromosomes and the interactions between homologue genes (Liu *et al.*, 2015).

*Brassica napus* is widely cultivated and is the world’s second leading crop source of vegetable oil (after soybean) for human consumption (Fu, 2004; Meyer, 2009). However, *B. napus* is highly susceptible to B deficiency (Marschner, 1995). To address this problem, borate fertilizers have been applied to soils with low B abundance (Wang *et al.*, 2007); however, borate rock is a depleting and non-renewable mineral resource. Moreover, among the essential mineral nutrients, boron has the narrowest margin in soil concentrations between deficiency and toxicity (Goldberg, 1997). Thus, the identification of *B. napus* genotypes with varying B efficiencies and cloning the genes regulating B efficiency is a prerequisite for breeding B-efficient rapeseed germplasm resources for use on B-deficient soils, which represents the most sustainable and environmentally friendly strategy for the agricultural industry to address the problem.

The boric acid influx channels and B efflux transporters in Arabidopsis, including AtBOR1 (Takano *et al.*, 2002), AtNIP5;1 (Takano *et al.*, 2006), and AtNIP6;1 (Tanaka *et al.*, 2008), have been identified as indispensable for efficient B uptake and translocation under B deficiency (Miwa and Fujiwara, 2010). However, in *B. napus* few of these genes have been finely mapped, except *qBEC-A3a* (Hua *et al.*, 2016), although QTLs for B efficiency have been characterized using several mapping populations (Xu *et al.*, 2001; Zhao *et al.*, 2008, 2012; Zhang *et al.*, 2014).

As a result of the rapid development of low-cost next-generation sequencing (NGS) technology, whole-genome re-sequencing (WGS) and RNA-seq/digital gene expression (DGE) profiling have been widely employed to identify genomic variations (Choi *et al.*, 2015; Lee *et al.*, 2016) and genome-wide differentially expressed genes (DEGs) under specific conditions (Gelli *et al.*, 2014; Liao *et al.*, 2015). Although map-based or positional cloning is an extremely powerful and unbiased technique to identify candidate genes underlying the target traits, delimiting QTLs to small genomic intervals remains a time-consuming and labor-intensive process (Fridman *et al.*, 2004). The QTL-seq approach was first formulated for the rapid mapping of QTLs in rice by the WGS of bulk DNA with phenotypic extremities (Takagi *et al.*, 2013). Subsequently, QTL-seq has been successfully applied to the rapid mapping of QTLs or quantitative trait genes (QTGs) for diverse agronomic traits in plant species, including semi-dwarfism and salt tolerance in rice (Abe *et al.*, 2012; Takagi *et al.*, 2015), seed weight and pod number in chickpea (Das *et al.*, 2015, 2016; Singh *et al.*, 2016), fruit weight and locule number in tomato (Illa-Berenguer *et al.*, 2015), early flowering and subgynoecy in cucumber (Lu *et al.*, 2014; Bu *et al.*, 2016), and yield-related traits in *B. napus* (Fu *et al.*, 2015).

In this current research, we aimed to achieve the following three objectives: (i) to phenotypically discriminate between B-efficient and B-inefficient *B. napus* genotypes under B deficiency; (ii) to reveal genomic variations and transcriptional differences between B-efficient and B-inefficient genotypes under B deficiency; and (iii) to identify the candidate genes underlying B efficiency in allotetraploid rapeseed through combining analyses of QTL-seq and the DEGs. This research facilitates our understanding of the differential tolerance to B deficiency in *B. napus* genotypes, and provides novel insights into the rapid cloning of QTGs in diverse plant species with complex genomes.

## Materials and methods

### Plant materials

The B-efficient (B-deficiency-resistant) rapeseed genotype ‘Qingyou 10’ (‘QY10’) and the B-inefficient (B-deficiency-sensitive) genotype ‘Westar 10’ (‘W10’), were used to perform analyses of the phenotypic and physiological differences in response to B deficiency during vegetative and reproductive development. The leaves and roots of 10-d-old ‘QY10’ and ‘W10’ seedlings exposed to B deficiency were subjected to DGE profiling in order to identify genome-wide DEGs. The ‘QY10’, ‘W10’, B-efficient and B-inefficient pools of the doubled haploid (DH) lines derived from ‘QY10’ and ‘W10’ were subjected to WGS to identify genomic variations and delineate the QTLs or genes underlying B efficiency. Using a hydroponic culture system, the plants were cultivated in an illuminated chamber for 10 d, and 25 μM and 0.25 μM B were used as the high and low B conditions, respectively. Using a pot culture system (see Hua *et al.*, 2016), plants were grown under high (1.0 mg per kg soil) or low (0.25 mg per kg soil) B conditions for the whole life cycle. The B efficiency coefficient (BEC) was defined according to Zhang *et al.* (2014) as follows: BEC = total dry weight (low B)/total dry weight (high B), or BEC = seed weight (low B)/seed weight (high B).

### Microscopy analysis

The roots of seedlings cultivated under the hydroponic culture system were imaged using a scanner, followed by determination of the total root length, root volume, and root surface area using the root image analysis software WinRHIZO Pro (Regent Instruments, QC, Canada). The length of the non-root-hair zones (NRHZs) in root tips with 10 replicates was quantified using ImageJ (http://rsb.info.nih.gov/ij/). Root hairs of fresh seedlings were examined using an Olympus SZX16 stereoscopic microscope (Olympus, Tokyo, Japan). The pattern of accumulation of reactive oxygen species (ROS) in the root tips was detected using dihydroethidium (DHE) (Oiwa *et al.*, 2013). For this purpose, the seedlings were incubated with 10 μM DHE for 30min in the dark. Subsequently, the roots were observed for ethidium fluorescence with a fluorescent microscope (Nikon Eclipse 80i; Nikon, Tokyo, Japan) equipped with a 510–560-nm excitation filter and a 590-nm barrier filter. Mature pollen grains (PGs) were stained with 1% acetocarmine to detect viability using the Nikon Eclipse 80i fluorescent microscope.

Pieces of juvenile leaves (approximately 1mm^2^) from the fresh seedlings were subjected to transmission electron microscopy (TEM) (H-7650; Hitachi, Tokyo, Japan) to characterize differences in cell morphologies, plasma membranes (PMs) and cell walls (CWs). At the full-blossom stage, anthers and stigmas were isolated from the stamens and pistils, respectively, and subjected to scanning electron microscopy (SEM) (JSM-6390/LV; JEOL, Tokyo, Japan) to characterize the PGs and mastoids.

### Quantification of lipid peroxidation and B accumulation

Lipid peroxidation was evaluated by determining the malondialdehyde (MDA) concentration in the leaves and roots of fresh seedlings as described by Bejaoui *et al.* (2016). Extraction of B in plant samples was performed according to Zhang *et al.* (2014), and then B was quantified by inductively coupled plasma mass spectrometry (ICP-MS, NexION^TM^ 350X; PerkinElmer, Massachusetts, USA).

### Whole-genome re-sequencing

An Illumina HiSeq 2000 system (read length = 100bp) (Illumina Inc., San Diego, CA, USA) was used to perform WGS to distinguish the genomic variations (including single nucleotide polymorphisms, SNPs, and insertions/deletions, InDels) between ‘QY10’ and ‘W10’, which generated a total of 40 Gb of data. To construct B-efficient and B-inefficient bulk DNA, the doubled haploid (DH) population comprising 190 lines derived from ‘QY10’ and ‘W10’, was subjected to B-efficiency assessment through an integrated analysis of B-deficiency symptoms and total dry biomass under the hydroponic culture system. ‘B-efficient’ plants were assumed to be higher in total dry weight or seed yield and without obvious B-deficiency symptoms when grown under B-deficient conditions compared to ‘B-inefficient’ plants. Based on the B efficiency assessment, individuals representing the two outermost ends of the normal frequency distribution curve of B efficiency were selected from the DH population of ‘QY10’ × ‘W10’ for QTL-seq analyses. After isolation and quantification of genomic DNA and the pooling of equal concentrations of DNA to constitute the B-efficient (BE) and B-inefficient (BinE) bulk samples, we used an Illumina HiSeq 3000 platform (read length = 150bp) (Illumina Inc., San Diego, CA, USA) to perform WGS. The high-quality homozygous SNPs between the BE and BinE bulk samples were further structurally identified and functionally annotated with the reference genome.

### Identification of differentially expressed genes through digital gene expression profiling

Leaves and roots of ‘QY10’ and ‘W10’ seedlings that had been exposed to B deficiency for 10 d were subjected to DGE profiling. The total RNA of each sample was subsequently sequenced on an Illumina Hiseq 2500 platform (San Diego, CA, USA) to generate 50-bp single-end (SE) reads.

High-quality clean reads were mapped to the *B. napus* ‘Darmor-*bzh*’ transcriptome reference, and then the mRNA abundances of the unigenes, which were identified by TopHat (http://ccb.jhu.edu/software/tophat/index.shtml) and Cufflinks (http://cole-trapnell-lab.github.io/cufflinks/) (Trapnell *et al.*, 2012), were normalized by the fragments per kilobase of exon model per million mapped reads (FPKM) (Trapnell *et al.*, 2010). The DEGs were defined as genes with a *P*-value and false discovery rate (FDR) less than 0.05 (Secco *et al.*, 2013). Multiexperiment Viewer (MeV; http://www.tm4.org/mev.html) (Eisen *et al.*, 1998) was used to delineate heat maps based on the DGE results. Gene ontology (GO) analyses of DEGs were performed using the PANTHER classification system (http://www.pantherdb.org/data/) (Mi *et al.*, 2005).

### QTL-seq analysis of candidate QTLs or genes for B efficiency in allotetraploid rapeseed

A well-established QTL-seq approach relying on the evaluation of the SNP-index and Δ(SNP-index) (Takagi *et al.*, 2013) was slightly modified to scan major QTLs for B efficiency in *B. napus* following the recommended parameters (Fu *et al.*, 2015). The SNP-index for each SNP position was calculated for both the bulk samples according to Abe *et al.* (2012) using the formula:

SNP-index (at a position) = Count of alternate base / Count of reads aligned

Based on the SNP index, the Δ(SNP index) was estimated as described by Takagi *et al.* (2013) using the modified formula:

Δ(SNP-index) = SNP-index (BE bulk) − SNP-index (BinE bulk)

A sliding-window approach with a 1-Mb window size and 10-kb increment was utilized to measure the average distribution of Δ(SNP-index) of the SNPs physically mapped across the *B. napus* ‘Darmor-*bzh*’ genome in a given genomic interval.

### Validation of the DGE results by real-time quantitative PCR

Real-time quantitative PCR (RT-qPCR) assays (primer sequences shown in Supplementary Table S1 at *JXB* online) were used to verify the DGE results according to a previously described protocol (Hua *et al.*, 2016).

### Statistical analysis and submission of sequencing data

Fisher’s least-significant difference (LSD) test (*P*-value <0.05) was performed for the analysis of all statistical tests using the software Statistical Product and Service Solutions 17.0 (SPSS, Chicago, IL, USA). All the sequencing data of the WGS and DGE profiling were submitted to the National Centre for Biotechnology Information (NCBI) (http://www.ncbi.nlm.nih.gov/) with the Bioproject PRJNA340053.

## Results

### Differential vegetative responses to B deficiency between B-efficient and B-inefficient genotypes

**Fig. 1.**
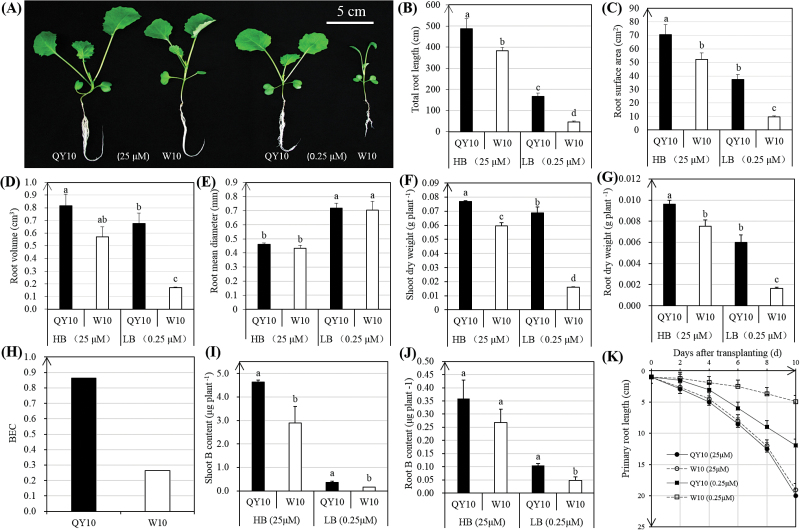
Vegetative performance of the B-efficient genotype ‘QY10’ and the B-inefficient genotype ‘W10’ seedlings. (A) Growth performance of ‘QY10’ and ‘W10’ grown hydroponically under high (25 μM) and low (0.25 μM) B conditions for 10 d. (B–E) Root-related indices of the seedlings: total root length (B), root surface area (C), root volume (D), and root mean diameter (E). (F, G) The shoot (F) and root (G) dry weights of the seedlings. (H) B efficiency coefficient (BEC) of ‘QY10’ and ‘W10’, where BEC = total dry weight (0.25 μM)/total dry weight (25 μM) (Zhang *et al.*, 2014). (I, J) B content in the shoots (I) and roots (J). (K) Primary root elongation rate. For (A–G) the statistical analyses were conducted using all the performance data of the B-efficient and B-inefficient genotypes under high and low B conditions; for (I) and (J) statistical analyses of the performance data obtained under high and low B were conducted, respectively. Data presented are the means (*n*=3), and error bars denote the standard deviations. Different letters indicate significant differences at *P*-value <0.05. (This figure is available in colour at *JXB* online.)

In order to assess the differential vegetative resistances of ‘QY10’ and ‘W10’ to B deficiency, plants were subjected to a hydroponic culture system. Under B limitation, the seedlings of ‘W10’ exhibited evident developmental defects in the vegetative organs, including curved juvenile leaves and retarded primary roots (Fig. 1A), all of which were characteristic of B-deficiency symptoms. A microscopic analysis of the roots showed that ‘QY10’ developed greater total root length, root surface area, and root volume than ‘W10’ (Fig. 1B–D), while the root mean diameters of both ‘QY10’ and ‘W10’ were significantly increased with B deficiency (Fig. 1E). Under B limitation, the shoot and root dry weights of ‘QY10’ were markedly higher than ‘W10’ (Fig. 1F, G), which contributed to the larger BEC for ‘QY10’ than ‘W10’ (Fig. 1H). The B content in the shoots was much higher than in the roots for both lines, and the B content in the shoots and roots of ‘QY10’ was considerably higher than in ‘W10’ (Fig. 1I, J), indicating the stronger capability of ‘QY10’ to accumulate B under deficiency conditions. The hydroponic culture also showed that ‘QY10’ possessed a higher elongation rate of the primary roots than ‘W10’ when they were exposed to B deficiency (Fig. 1K).

**Fig. 2.**
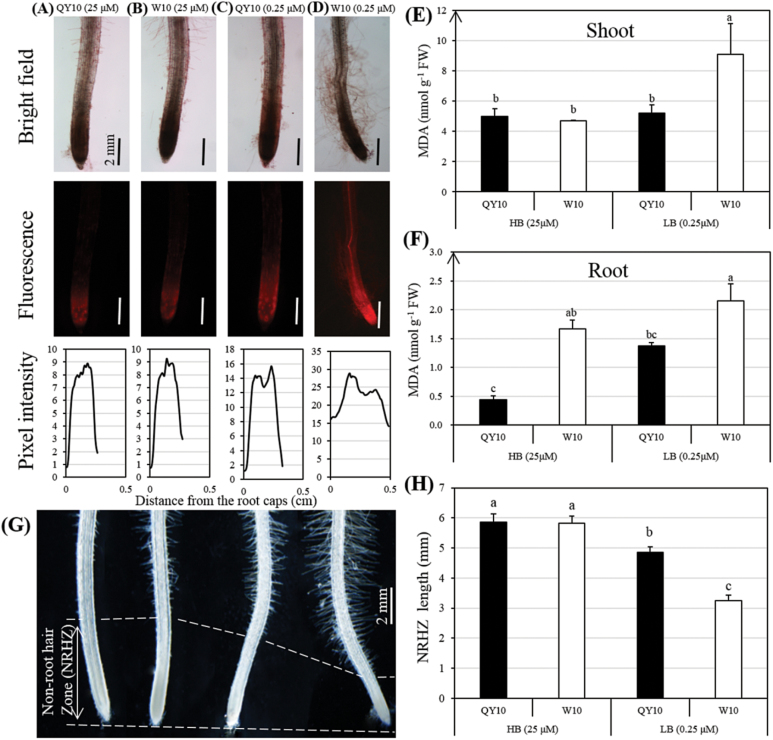
Reactive oxygen species (ROS)/malondialdehyde (MDA) detection and characterization of root hairs in root tips of the B-efficient genotype ‘QY10’ and the B-inefficient genotype ‘W10’ seedlings under a hydroponic culture system. (A–D) Dihydroethidium (DHE) staining in the roots of the seedlings grown under high (25 μM) and low B (0.25 μM) conditions for 10 d. The images show representative individuals of two independent experiments, with at least five seedlings examined for each experiment. The fluorescence intensity was scanned using the ImageJ plot profile (https://imagej.nih.gov/ij/). The *x*-axis is distance (cm) from the root caps, and the *y*-axis is relative pixel intensity. (E, F) MDA levels in the shoots (E) and roots (F) of ‘QY10’ and ‘W10’. Data presented are the means (*n*=3), and error bars denote the standard deviations. (G, H) Comparative analyses of the density and length of root hairs (G) and the length of non-root-hair zones (NRHZs) (G, H) between ‘QY10’ and ‘W10’. The root images in (G) correspond to the data presented in (H), which are the means (*n*=10), and error bars denote the standard deviations. The significance level was set at a *P*-value < 0.05. (This figure is available in colour at *JXB* online.)

It has been established that ROS are involved in the inhibition of root elongation caused by B deficiency (Camacho-Cristóbal *et al.*, 2015). Therefore, DHE staining was used to identify the differential inhibition effect of ROS on the root elongation of ‘QY10’ and ‘W10’. Interestingly, B deficiency caused a greater increase in red fluorescence in the root tips of ‘W10’ than ‘QY10’ (Fig. 2A–D), which suggested the possible involvement of ROS production in the differential response to B deprivation. This hypothesis was supported by the fact that genes related to antioxidant enzymes (including POD, SOD, CAT, APX) presented higher transcript levels in the roots of ‘QY10’ than ‘W10’, while the expression patterns in the shoots were generally opposite to the ones in the roots (Supplementary Fig. S1). Furthermore, the over-accumulated MDA in ‘W10’ showed that it suffered from more severe lipid peroxidation in both the shoots and roots than ‘QY10’ (Fig. 2E, F), which might cause greater damage to the integrity of cell membranes. A stereomicroscopic analysis showed that B deficiency inhibited the elongation of non-root-hair zones (including the root caps, meristem zones, and elongation zones) (Fig. 2G, H) of both ‘QY10’ and ‘W10’, which was also supported by the findings of previous studies (Abreu *et al.*, 2014; Li *et al.*, 2015). However, low B triggered a greater increase in the density and length of root hairs of ‘W10’ (Fig. 2G) due to its higher susceptibility to B deficiency.

**Fig. 3.**
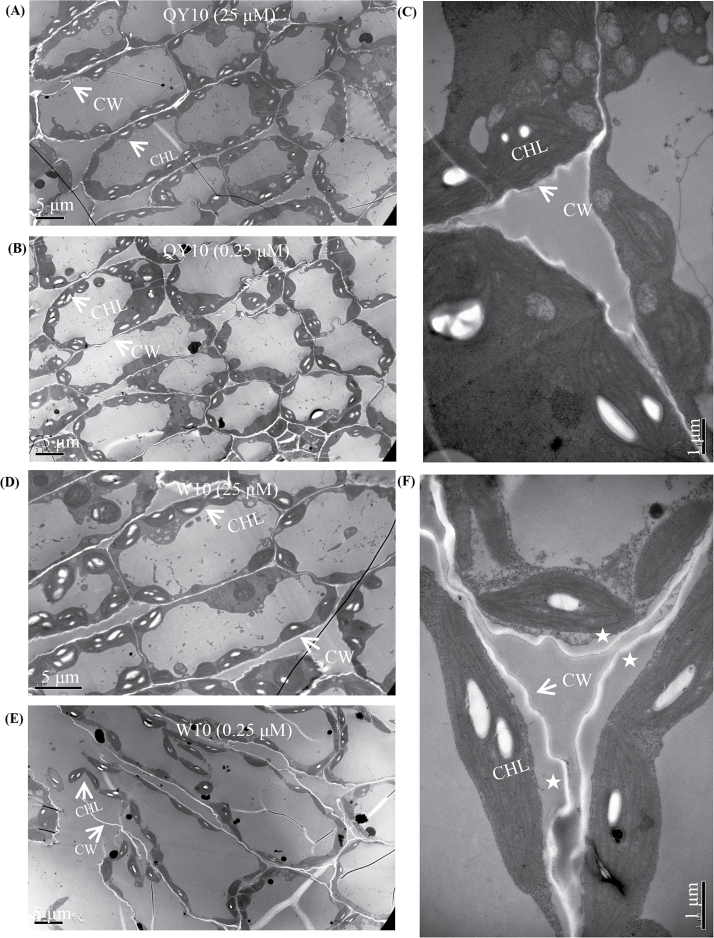
Transmission electron microscopy (TEM) analysis of the juvenile leaves of the B-efficient genotype ‘QY10’ and the B-inefficient genotype ‘W10’ seedlings. (A, B) Low-magnification view of the chloroplasts (CHLs) arrayed along plasma membranes (PMs) and the cell morphologies of ‘QY10’ under 25 μM (A) and 0.25 μM (B) B conditions. (C) Close-up view of PMs and cell walls (CWs) of ‘QY10’ under 0.25 μM B. (D, E) Low-magnification view of the CHLs arrayed along PMs and cell morphologies of ‘W10’ under 25 μM (D) and 0.25 μM (E) B conditions. (F) Close-up view of PMs and CWs of ‘W10’ under 0.25 μM B. Asterisks denote plasmolysis in the cells, which was indicated by shrunken cells and PMs.

To examine the cellular effects underlying the vegetative morphological differences between ‘QY10’ and ‘W10’, we analysed the ultrastructure of juvenile leaves at the seedling stage using TEM. Under high B (25 μM), cells of both ‘QY10’ and ‘W10’ appeared structurally intact, and the chloroplasts were arrayed in an orderly manner along the plasma membranes (PMs) (Fig. 3A–D). However, under B deficiency (0.25 μM), although the cell morphology of ‘QY10’ remained similar to that under high B (Fig. 3B, C), ‘W10’ showed severely detached cells and plasmolysis, including loosened and swollen CWs, shrunken cells, and PMs (Fig. 3E, F), which coincided with its severe lipid peroxidation as indicated by MDA, as noted above (Fig. 2E, F). Both of these factors showed greater susceptibility of the ‘W10’ plants’ to B deficiency.

### Differential reproductive responses to B deficiency between B-efficient and B-inefficient genotypes

**Fig. 4.**
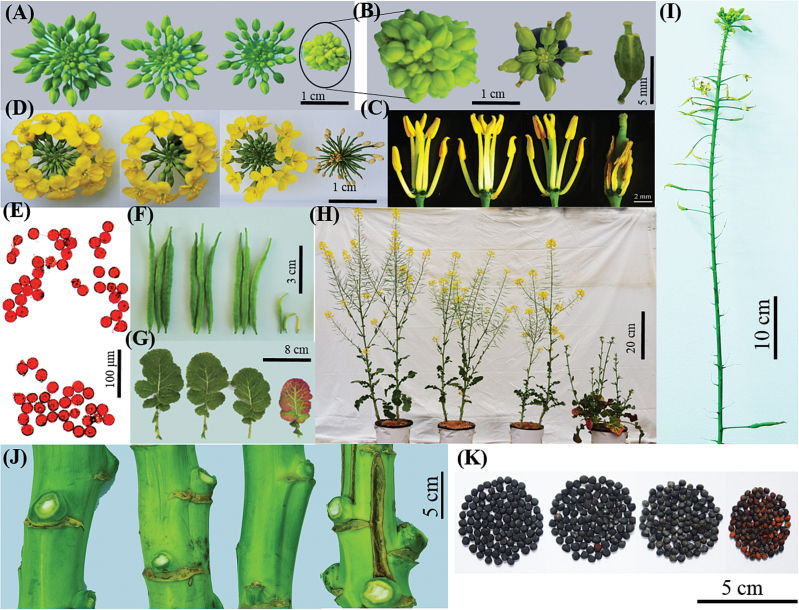
Reproductive growth of the B-efficient genotype ‘QY10’ and the B-inefficient genotype ‘W10’ under a pot culture system. Images are left to right (A–K except B, E, and I): ‘QY10’ (1.0 mg B per kg soil), ‘W10’ (1.0 mg B per kg soil), ‘QY10’ (0.25 mg B per kg soil) and ‘W10’ (0.25 mg B per kg soil). (A, B) Overview of the floral buds of ‘QY10’ and ‘W10’ (A), and close-up view of the floral buds of ‘W10’ under B deficiency (0.25 mg B per kg soil) conditions (B); (C) floral phenotypes; (D) overview of the flowers; (E) viabilities of pollen grains of ‘QY10’ (upper panel) and ‘W10’ (lower panel) under B deficiency (0.25 mg B per kg soil), which were detected by 1% acetocarmine; (F) morphologies of the siliques; (G) size and colour of the leaves; (H) overview of mature plants; (I) performance of the main inflorescence of ‘W10’ under B deficiency (0.25 mg B per kg soil) conditions; (J) morphologies of the stems; (K) seed size. (This figure is available in colour at *JXB* online.)

To further determine the differential responses of ‘QY10’ and ‘W10’ to B deficiency in reproductive development, they were grown in a pot culture system. Under B limitation, the floral buds of ‘W10’ displayed protruding stigmas and shorter anther filaments with abnormal morphologies compared with ‘QY10’ (Fig. 4A–C), leading to a physical separation of the stamens from the stigmas, which posed a greater threat to self-pollination. Even more detrimental than this, ‘W10’ gradually presented floral abscission (Fig. 4D). Although we could not distinguish obvious differences in the viabilities of the PGs between ‘QY10’ and ‘W10’ under B deficiency (Fig. 4E), the B-inefficient ‘W10’ line presented reduced fertility, reflected by seedless and/or short siliques (Fig. 4F). ‘W10’ also showed more severe developmental defects in the plant organs, including discoloured leaves (Fig. 4G), severe bushiness reflected by reduced stature or loss of apical dominance (Fig. 4H, Supplementary Fig. S2A), fewer siliques at the main inflorescence (Fig. 4I, Supplementary Fig. S2B), and split stems (Fig. 4J). After long-term exposure to B deficiency, ‘W10’ produced smaller seeds than ‘QY10’ (Fig. 4K), which was also reflected by a lower seed yield (Supplementary Fig. S2C) and 1000-seed weight (Supplementary Fig. S2D). Of special interest, we also found that ‘QY10’ possessed a higher BEC and oil content than ‘W10’ (Supplementary Fig. S2E, F). Thus, ‘QY10’ represents an elite germplasm resource that can be used for B-efficient rapeseed breeding in the agricultural industry.

**Fig. 5.**
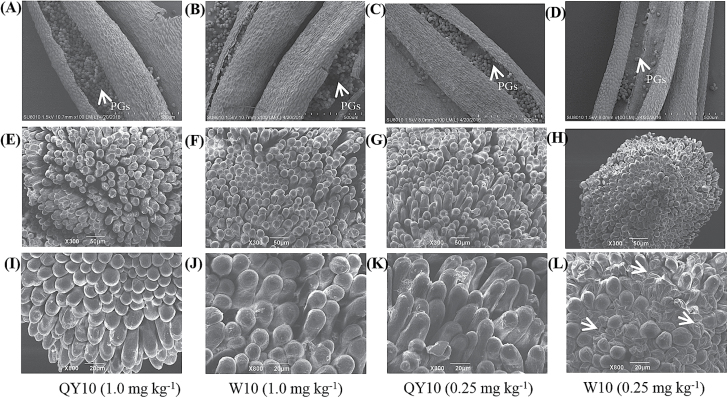
Scanning electron microscopy (SEM) analysis of anthers and stigmas of the B-efficient genotype ‘QY10’ and the B-inefficient genotype ‘W10’, which were grown under high (1.0 mg per kg soil) and low (0.25 mg per kg soil) B conditions in a pot culture system. (A–D) Overview of the anthers of ‘QY10’ and ‘W10’. Pollen grains (PGs) are indicated by arrows. (E–H) Low-magnification view of the stigma morphologies of ‘QY10’ and ‘W10’; (I–L) close-up view of the stigma morphologies of ‘QY10’ and ‘W10’. (L) The over-crowded, collapsed, and shrivelled mastoids on the top of the stigmas of ‘W10’ under B deficiency are indicated by arrows.

Given the great differences in the fertilities of ‘QY10’ and ‘W10’ (Fig. 4), we paid close attention to the performance of the stamens and pistils. SEM analysis showed that the number of pollen grains in the anthers of ‘W10’ was significantly reduced compared with ‘QY10’ under B deficiency (Fig. 5A–D). Moreover, the mastoids on the top of the stigmas of ‘W10’ appeared to be over-crowded, collapsed, and shrivelled compared to the corresponding structures in ‘QY10’ when suffering from B deficiency (Fig. 5E–L), which caused great difficulty in fertilization and ultimately led to widespread sterility in ‘W10’.

### Identification of genomic variations between B-efficient and B-inefficient genotypes through whole-genome re-sequencing

**Fig. 6.**
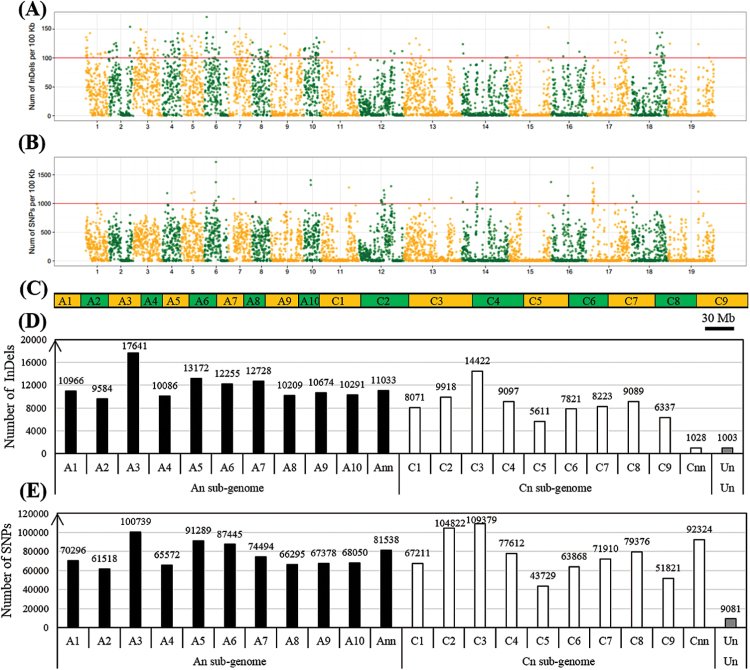
Distribution and number of genome-wide single nucleotide polymorphisms (SNPs) and insertions/deletions (InDels) between the B-efficient genotype ‘QY10’ and the B-inefficient genotype ‘W10’. (A, B) Distribution of InDels (A) and SNPs (B). The *x*-axis represents the *B. napus* chromosome sizes (Mb) while the *y*-axis represents the number of SNPs or InDels present at that point on each chromosome. (C) Graph delineating *B. napus* chromosome sizes; (D, E) number of InDels (D) and SNPs (E) on each chromosome. ‘A_nn_’ or ‘C_nn_’ represents the genome scaffolds anchored to the A_n_ or C_n_ subgenome but not anchored to specific chromosomes; ‘U_n_’ represents the genome scaffolds whose locations are unknown. (This figure is available in colour at JXB online.)

To identify the genomic variations between the B-efficient and B-inefficient genotypes, we performed WGS of ‘QY10’and ‘W10’, generating a total of 20 Gb (18×) data for each of them, which revealed 218 755 InDels unevenly distributed over the *B. napus* genome (A1–A10, C1–C9) (Fig. 6A–C), ranging from 5611 (chr. C5) to 17 641 (chr. A3) with an average of 11 566 InDels on each chromosome (Fig. 6D). The lengths of InDels ranged from mono-nucleotide to 18-nucleotide, and the frequency was negatively correlated with the number of nucleotides (Supplementary Fig. S3A). Mono-nucleotide InDels (135 101, 61.8%) were the most frequent type, followed by di- (34 588, 15.8%) and tri-nucleotides (17 891, 8.17%) (Supplementary Fig. S3A).

In total, 1 605 747 SNPs were identified across the 19 chromosomes of *B. napus* (Fig. 6B, C), ranging from 43 729 (chr. C5) to 109 379 (chr. C3) with an average of 84 513 SNPs on each chromosome (Fig. 6E). The frequency of SNPs on each chromosome varied from 1 per 960bp (chr. C5) to 1 per 251bp (chr. A5), with an average value of 1 per 465bp, and the nucleotide diversity *π* (average number of SNPs per nucleotide) ranged from 1.04×10^–3^ (chr. C5) to 3.97×10^−3^ (chr. A5), with an average value of *π*=2.15×10^−3^. The SNPs detected were categorized into two groups, transitions (A/G and C/T; Ts) and trans-versions (A/C, A/T, C/G, and G/T; Tv), based on the nucleotide variations between ‘QY10’ and ‘W10’. Among the 1 605 747 SNPs, 920 229 (57.3%) belonged to the transition type, which was more common than the trans-versions (685 518, 42.7%) (Supplementary Fig. S3B). With regards to the transitions, the frequency of A/G transitions (460 763, 28.7%) was similar to the frequency of C/T (459 466, 28.6%); however, among the trans-versions, the frequency of the A/C sub-type (212 697, 13.2%) showed it to be the most common and C/G to be the least (112 226, 6.90%) (Supplementary Fig. S3B). We then used the annotations of the reference ‘Darmor-*bzh*’ genome to examine the distribution of SNPs and InDels within various genomic features. Overall, a similar distribution of SNPs and InDels was observed (Supplementary Fig. S3C). Only 11.6% of total SNPs and 11.1% of total InDels were detected in genic regions, whereas a significant proportion of SNPs (87.3%) and InDels (87.6%) were detected in 2-kb upstream (promoter), 1-kb downstream, and other inter-genic regions. Further, we analyzed the effect of SNPs on amino acid substitution. Of the SNPs present in coding sequence regions, a smaller fraction (~40%) was detected to be of the non-synonymous than the synonymous type (~60%) (Supplementary Fig. S3D).

### Global identification of differentially expressed genes between B-efficient and B-inefficient genotypes under B deficiency

**Fig. 7.**
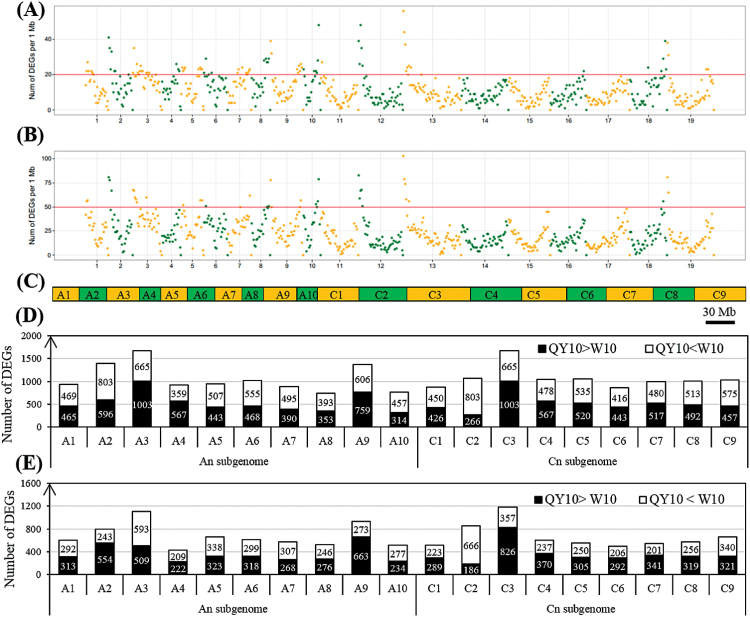
Distribution and number of genome-wide differentially expressed genes (DEGs) between the B-efficient genotype ‘QY10’ and the B-inefficient genotype ‘W10’. (A, B) Distribution of the DEGs in the shoots (A) and roots (B). The *x*-axis represents the chromosome size (Mb) while the *y*-axis represents the number of SNPs or InDels at that point on each chromosome; (C) graph delineating the *B. napus* chromosome sizes; (D, E) number of DEGs in the shoots (D) and roots (E). ‘QY10 > W10’ indicates that the transcript levels of DEGs are higher in ‘QY10’ than in ‘W10’, and vice versa. (This figure is available in colour at *JXB* online.)

A DGE profiling strategy was employed to identify genome-wide transcriptional differences between the B-efficient and B-inefficient lines under B deficiency. A total of 155 million clean reads were obtained with an average of 13 million reads (0.65 Gb data) for each sample (Supplementary Table S2). In total, 21 743 and 14 343 DEGs between the two lines were identified in the shoots and roots, respectively, which were unevenly distributed on the 19 chromosomes of *B. napus* (Fig. 7A–C). Specifically, in the shoots, there were more DEGs (11 127, 11.0%) with higher expression levels in ‘W10’ than in ‘QY10’ (10,616, 10.5%) (Fig. 7D, Supplementary Fig. S4A). In contrast, in the roots, ‘QY10’ had more genes (7900, 7.82%) with higher mRNA abundances than ‘W10’ (6443, 6.38%) (Fig. 7E, Supplementary Fig. S4A), including the transporters principally responsible for nutrient uptake and transport (Supplementary Fig. S4B), which highlights the pivotal role of roots in regulating B efficiency between *B. napus* genotypes.

A gene ontology (GO) enrichment analysis of functional significance allowed us to distinguish major biological functions of the DEGs between ‘QY10’ and ‘W10’. The differentially expressed transcripts could be grouped into the following four categories: molecular function (MF), cellular component (CC), biological process (BP), and protein class (PC) (Supplementary Fig. S5). Regardless of the shoots or the roots, the most highly enriched GO term for CC was macromolecular complex, while catalytic activity was the most enriched in the MF category. In the BP annotations, response to stimuli was the most enriched. In PC, transporter, transferase, oxidoreductase, and kinase were the four strongest GO enrichments.

**Fig. 8.**
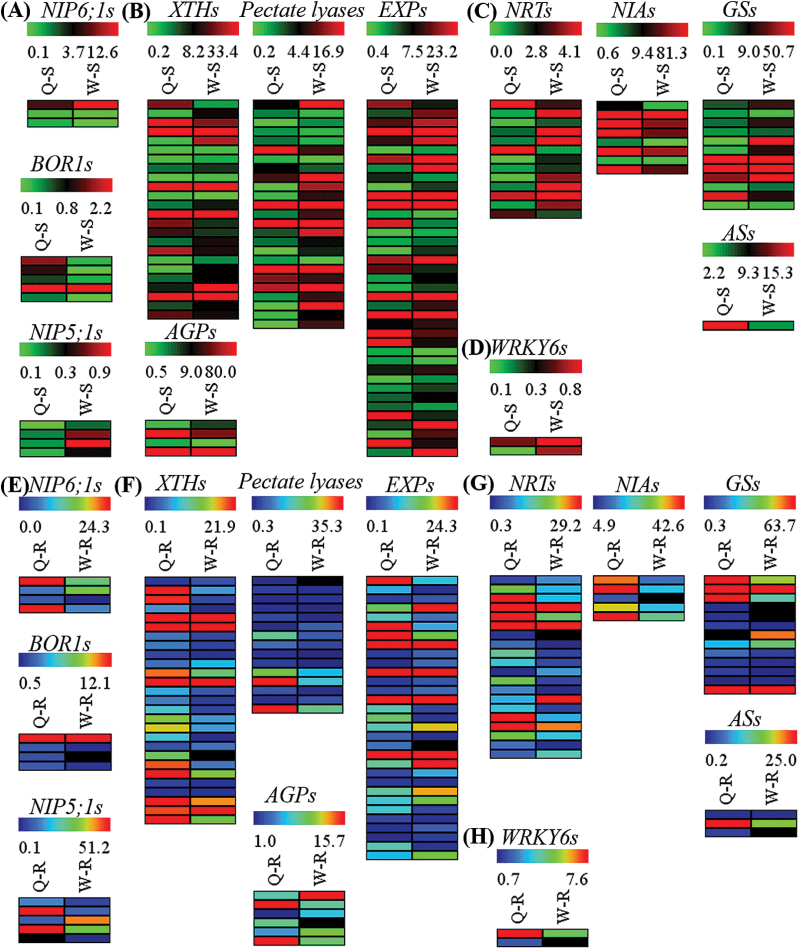
Expression profiles of key differentially expressed genes (DEGs) between the B-efficient genotype ‘QY10’ and the B-inefficient genotype ‘W10’. DEGs in the shoots (A–D) and roots (E–H), including the DEGs related to B uptake and transport (*BOR1*, *NIP5;1*, and *NIP6;1*) (A, E), the DEGs related to the maintenance of cell wall and membrane function (B, F), the DEGs related to nitrate uptake, transport, and assimilation (C, G), and the DEGs of *WRKY6s* (D, H). XTHs, xyloglucan endo-trans-glycosylase/hydrolases; EXPs, expansins; AGPs, arabinogalactan-proteins; NRTs, high-affinity nitrate transporters; NIAs, nitrate reductases; GSs, glutamine synthetases; ASs, asparagine synthetases. Q, ‘QY10’; W, ‘W10’; S, shoot; R, root. (This figure is available in colour at *JXB* online.)

Given the considerable differences in B accumulation between ‘QY10’ and ‘W10’ (Fig. 1I, J), we compared the transcript levels of *BnaNIP5;1*s, *BnaBOR1*s, and *BnaNIP6;1*s, which might also function as the transporters responsible for B uptake, transport, and distribution in *B. napus* under B deficiency. In the shoots, few significant differences in the expression profiles of *BnaNIP5;1*s and *BnaBOR1*s were observed between the two genotypes, whereas *BnaNIP6;1*s were more highly expressed in the shoots of ‘W10’ than ‘QY10’ (Fig. 8A). However, the transcript levels of B transporter genes in the roots of ‘QY10’ were obviously higher than for ‘W10’ (Fig. 8E), which may be the determinants of the greater accumulation of B in ‘QY10’ than in ‘W10’ (Fig. 1I, J).

Given that the PMs and CWs of ‘W10’ were much more severely impaired and swollen than in ‘QY10’ under B deficiency (Fig. 3H, I), the DEGs related to the maintenance of CW and membrane function (XTHs, expansins, pectate lyases, and AGPs), which have been identified as being indispensable for CW loosening (Camacho-Cristóbal *et al.*, 2011), were subjected to expression profile analyses. More DEGs related to expansins possessed higher transcript levels in ‘W10’ irrespective of the shoots (Fig. 8B) or the roots (Fig. 8F); however, more genes related to XTHs and pectate lyases presented higher expression profiles in the roots of ‘W10’ than ‘QY10’ (Fig. 8F), although their transcript profiles in the shoots were highly similar between the two genotypes (Fig. 8B). In contrast, more genes related to AGPs showed higher expression profiles in the roots of ‘QY10’ than ‘W10’ (Fig. 8F), although their transcript profiles in the shoots were highly similar between the two genotypes (Fig. 8B). Taken together, the genes related to maintenance of CW and membrane function reacted differentially to B deficiency between B-efficient and B-inefficient genotypes.

Previous studies have shown that B deficiency affects the transcript levels of genes related to nitrate uptake, transport, and assimilation (Camacho-Cristóbal *et al.*, 2011), so it was assumed that the genes related to nitrate assimilation in the *B. napus* genotypes might present distinct expression profiles. To examine this hypothesis, we delineated transcript profiles of the genes related to nitrate assimilation, which included high-affinity nitrate transporters (*NRT*s), nitrate reductases (*NIA*s), glutamine synthetases (*GS*s), and asparagine synthetases (*AS*s). Generally, there existed considerable similarities between the expression profiles of these genes in the shoots of ‘QY10’ and ‘W10’ (Fig. 8C). However, without exception, higher expression levels of the four genes were detected in the roots of ‘QY10’ compared with ‘W10’ (Fig. 8G). In addition, the transcript levels of homologue genes of *AtWRKY6*, the first low-B-induced transcription factor found to be essential for normal root growth under low-B conditions in Arabidopsis (Kasajima *et al.*, 2010), were generally higher in the roots of ‘QY10’ than ‘W10’, which might suggest its involvement in the differential responses to B deficiency between *B. napus* genotypes.

### Identification of candidate genes underlying B efficiency through the integrated analyses of DEGs and QTL-seq

In order to refine the genomic regions harbouring QTLs for B efficiency, QTL-seq was conducted to compare the SNP profiles among the B-efficient ‘QY10’, B-inefficient ‘W10’, and the B-efficient and B-inefficient DH line pools on a genome-wide scale. Based on B-efficiency assessment, each of the DH line pools consisted of 22 plants selected from the 190-line DH population with 61% coefficient of variation, the dry biomasses of which ranged from 0.0085 to 0.24g per plant (Supplementary Fig. S6).

**Fig. 9.**
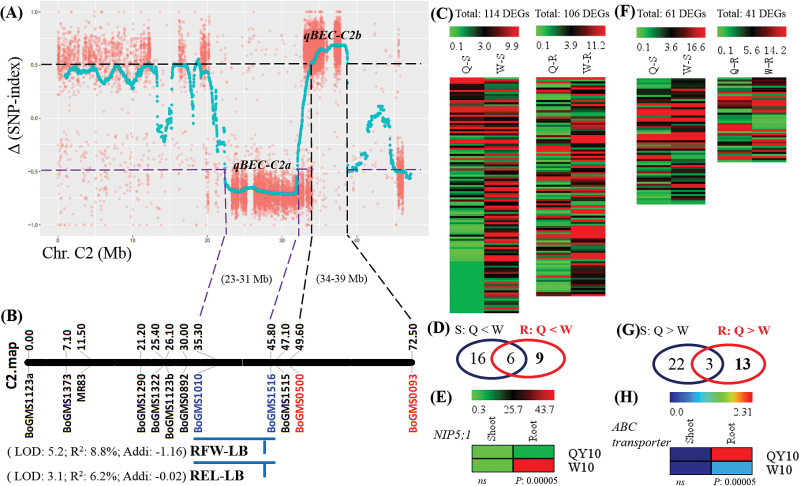
Digital gene expression (DGE) -assisted QTL-seq analysis for the identification of candidate genes underlying B efficiency in *B. napus*. (A) Two QTLs (*qBEC-C2a* and *qBEC-A3b*) for B efficiency identified by the whole-genome re-sequencing (WGS) of pools of DH lines derived from ‘QY10’ and ‘W10’. (B) QTLs for root fresh weight (RFW) and root elongation length (REL) under low B (LB, 0.25 μM) conditions, which were previously identified by Kou (2013). (C) Expression profiles of differentially expressed genes (DEGs) in the QTL *qBEC-C2a* region. (D) Venn diagram to screen the DEGs in the QTL *qBEC-C2a* region in the shoots and roots, all of which presented higher transcript levels in ‘W10’ than ‘QY10’. (E) Expression profile of *BnaC2.NIP5;1*. (F) Expression profiles of DEGs in the QTL *qBEC-C2b* region. (G) Venn diagram used to screen the DEGs in the QTL *qBEC-C2b* region in the shoots and roots, all of which presented higher transcript levels in ‘QY10’ than ‘W10’. (H) Expression profile of *BnaC2.ABCG21*. S, shoot; R, root; Q, ‘QY10’; W, ‘W10’; ‘Q > W’ indicates that the transcript levels of DEGs are higher in ‘QY10’ than in ‘W10’, and vice versa. (This figure is available in colour at *JXB* online.)

A total of 50 Gb (45×) data for each bulk DNA was generated from WGS. According to the definition of the SNP-index and Δ(SNP-index) in the QTL-seq analysis, we identified two genomic regions on chromosome C2 showing great differences in the indices of individual SNPs presenting differentiation between the B-efficient and B-inefficient pools, which may harbour QTLs for B efficiency (Fig. 9A). We designate these as *qBEC-C2a* and *qBEC-C2b*, the Δ(SNP-index) values of which were negative and positive, respectively. Our previous study had determined that negative and positive Δ(SNP-index) values represented QTLs contributed by the B-inefficient and B-efficient lines, respectively (Hua *et al.*, 2016). Thus, it was assumed that the *qBEC-C2a* and *qBEC-C2b* QTLs for B efficiency were contributed by ‘W10’ and ‘QY10’, respectively.

**Fig. 10.**
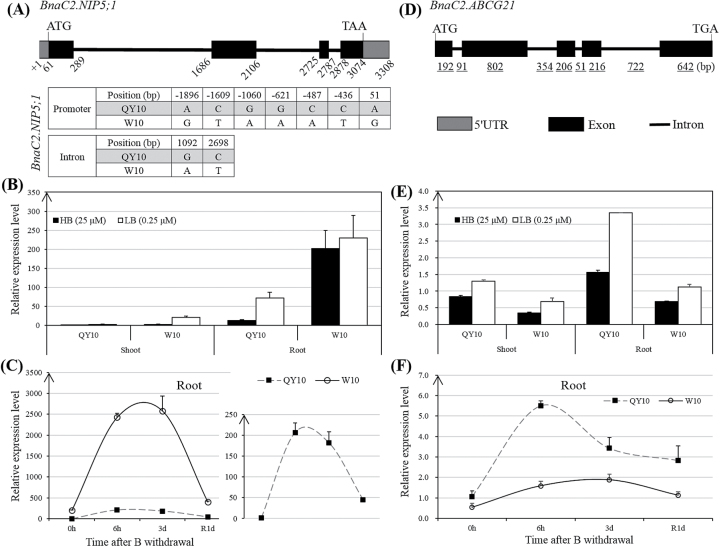
Gene structures and validation of *BnaC2.NIP5;1* and *BnaC2.ABCG21* expression levels by RT-qPCR. (A) Gene structures and allelic variations of *BnaC2.NIP5;1* between the B-efficient genotype ‘QY10’ and the B-inefficient genotype ‘W10’. The DNA polymorphisms of *BnaC2.NIP5;1* between ‘QY10’ and ‘W10’ were identified by whole-genome re-sequencing (WGS), and the transcription start site was defined by ‘+1’. (B, C) Validation of *BnaC2.NIP5;1* expression levels in the roots under long-term (B) and short-term (C) B deficiency conditions. (D) Gene structure of *BnaC2.ABCG21*; (E-F) validation of *BnaC2.ABCG21* expression levels in the roots under long-term (E) and short-term (F) B deficiency conditions. HB/LB, high (25 μM)/low (0.25 μM) B; R1d, B resupply for 1 d. Long-term B deficiency conditions: *B. napus* seedlings were cultivated for 20 d under 0.25 μM B conditions. Short-term B deficiency conditions: *B. napus* seedlings were cultivated for 10 d under 10 μM B and were then transferred to a solution without B supply. Data presented are the means (*n*=3), and error bars denote the standard deviations.

Compared with the QTL mapping results generated by traditional QTL scanning, the *qBEC-C2a* interval (23–31Mb) (Fig. 9A) was co-localized with a QTL cluster flanked by the simple sequence repeat (SSR) -based markers BoGMS1010 and BoGMS1516, where a QTL [LOD = 5.2; phenotypic variation explained (PVE)/*R*^2^ = 8.8%; additive effect = −1.16] for root fresh weight (RFW) and a QTL for root elongation length (REL), both of which were contributed by the B-inefficient parent ‘W10’, were identified under B deficiency conditions (Fig. 9B) (Kou, 2013). Obviously, the Δ(SNP-index) value was consistent with the QTL additive effect. Within the *qBEC-C2a* QTL region, there were 420 annotated genes from the *Brassica* Database (BRAD; http://brassicadb.org/brad/) (Cheng *et al.*, 2011). To examine the candidate genes controlling B efficiency within the *qBEC-C2a* region, we further performed analyses of the DEGs within the QTL interval. Among the 420 annotated genes, there were 114 and 106 genes differentially expressed (*P*<0.05) in the shoots and roots, respectively, between ‘QY10’ and ‘W10’ (Fig. 9C). According to a previous finding that B efficiency in *B. napus* is primarily controlled by the roots (Yang *et al.*, 2013), the search for candidate genes underlying *qBEC-C2a* was first concentrated on the DEGs in the roots. Previous studies have also established that the positive regulator genes (including *AtBOR1*, *AtNIP5;1*, *AtNIP6;1*, and *AtWRKY6*) play major roles in regulating B efficiency in Arabidopsis (Takano *et al.*, 2002, 2006; Tanaka *et al.*, 2008; Kasajima *et al.*, 2010) and that close relative relationships exist between Arabidopsis and *B. napus* (Chalhoub *et al.*, 2014). Thus, the search for candidate genes was preferentially focused on the DEGs with uniquely higher transcript levels in the roots of ‘W10’ compared to the roots of ‘QY10’. The resulting Venn diagram revealed that nine DEGs were identified as candidates (Fig. 9D, Supplementary Table S3), and, among them, more attention was paid to the *BnaC02g29210D* gene (Figs 9D, E and 10A, Supplementary Table S3) because it is highly homologous (92.8%) to *AtNIP5;1* (AT4G10380), which is an influx boric acid channel essential for efficient B uptake and normal plant development under B-limitation conditions (Takano *et al.*, 2006). Therefore, we refer to the *BnaA03g24370D* gene hereafter as *BnaC2.NIP5;1*. The WGS results revealed nine SNPs of *BnaC2.NIP5;1* between ‘QY10’ and ‘W10’, including seven SNPs in the promoter and two SNPs in the introns (Fig. 10A), that might be responsible for the differential transcript levels. Subsequently, the RT-qPCR assays validated that *BnaC2.NIP5;1* was preferentially expressed in the roots compared with the shoots irrespective of high- or low-B conditions (Fig. 10B), which was to the same as found for *AtNIP5;1* (Takano *et al.*, 2006). In addition, under B deficiency, the expression level of *BnaC2.NIP5;1* in the roots of ‘W10’ was strikingly elevated compared to the roots of ‘QY10’ (Fig. 10B, C), which was consistent with the DGE results (Fig. 9E). Moreover, under short-term B deficiency conditions, the abundance of *BnaC2.NIP5;1* mRNA was increased after B withdrawal and suppressed when B was resupplied (Fig. 10C), indicating that *BnaC2.NIP5;1* acted in a B-dependent manner similar to *AtNIP5;1*. Thus, *BnaC2.NIP5;1* was assumed to function as a B transporter and considered to be a prior candidate gene underlying the *qBEC-C2a* QTL for B efficiency.

Similarly, the above strategy was applied to identify candidate genes within the *qBEC-C2b* region. Among the 433 annotated genes, 61 and 41 genes were differentially expressed (*P*<0.05) between ‘QY10’ and ‘W10’ in the shoots and roots, respectively (Fig. 9F). The search for candidate genes underlying *qBEC-C2b* was concentrated on the DEGs with uniquely higher transcript levels in the roots of ‘QY10’ compared to the roots of ‘W10’. The resulting Venn diagram revealed that 13 DEGs were identified as candidates (Fig. 9G, Supplementary Table S4). Among them, particular attention was paid to *BnaC02g29210D* (hereafter referred to as *BnaC2.ABCG21*) (Fig. 9H, 10D) because it was highly homologous (87.7%) to an ATP-binding cassette (ABC) transporter gene *ABCG21*. *ABCG21* is a member of the ABC transporter gene superfamily (including A–G and I types), some of which have been established to drive the exchange of a wide range of compounds across many different biological membranes in terrestrial plants (Hwang *et al.*, 2016). Subsequently, the RT-qPCR results demonstrated that *BnaC2.ABCG21* was induced irrespective of long- or short-term B deficiency conditions (Fig. 10E), and its mRNA abundance was suppressed when B was resupplied (Fig. 10F), indicating that *BnaC2.ABCG21* also acted in a B-dependent manner.

## Discussion

### Whole-genome re-sequencing contributes to the identification of genomic variations between B-efficient and B-inefficient rapeseed genotypes

The availability of the complete *B. napus* (‘Darmor-*bzh*’) genome reference sequence (Chalhoub *et al.*, 2014) and the advancement of next-generation sequencing (NGS) technologies (including WGS and RNA-seq, among others) provide great opportunities to reveal the genetic diversity among various *B. napus* genotypes and for the genetic enhancement of oilseed rape.

In this study, a global analysis of genomic variations based on the WGS of two *B. napus* genotypes, ‘QY10’ (B-efficient, China) and ‘W10’ (B-inefficient, Canada), has led to the discovery of millions of SNPs and InDels across the *B. napus* genome. A total of 1 605 747 SNPs and 218 755 InDels were identified, with an average density of 14.2 SNPs per kb (Fig. 6) and high-density coverage (18×) across the entire *B. napus* genome, which could be utilized for analyses of genetic diversity, QTL mapping, marker-assisted breeding (MAB), positional cloning, comparative mapping, and association studies in *B. napus*. An analysis of the number of SNPs and InDels in ‘QY10’ and ‘W10’ showed that the observed number of these polymorphisms in each chromosome was significantly different from the expected number based on their physical sizes (Fig. 6), but it was positively correlated with the number of the DEGs on each chromosome (Fig. 6, 7).

Meanwhile, the ratio of transitions to trans-versions (Ts/Tv) was 1.3 (Supplementary Fig. S3), showing an upward bias towards transitions from the expected ratio of 0.5. This phenomenon, known as ‘transition bias’, has been reported in *B. napus* (Huang *et al.*, 2013) and other plant species, including soybean (Lee *et al.*, 2016) and rice (Subbaiyan *et al.*, 2012; Jain *et al.*, 2014). In addition, and surprisingly, the total numbers of SNPs (834 614) and InDels (128 879) in the A_n_ sub-genome were far larger than the total SNPs (762 052) and the InDels (88 873) in the C_n_ sub-genome (Fig. 6), although the assembled C_n_ sub-genome (525.8Mb) was obviously larger than the A_n_ sub-genome (314.2Mb) (Chalhoub *et al.*, 2014). The results agreed that the A_n_ sub-genome shows higher genome diversity than the C_n_ sub-genome (Huang *et al.*, 2013; Fletcher *et al.*, 2016). Meanwhile, non-random distributions of SNPs and InDels throughout the *B. napus* genome were common, i.e. the number of genomic variations was not positively correlated with the size of the chromosome (Fig. 6), as previously observed in the *Brassica* relative *Arabidopsis thaliana* (Feltus *et al.*, 2004).

### Digital gene expression profiling facilitates the identification of genome-wide differentially expressed genes between B-efficient and B-inefficient rapeseed genotypes

Transcriptome sequencing, including DGE profiling, is a revolutionary approach for the study of quantitative changes in transcript abundance on a genome-wide scale (Asmann *et al.*, 2009; Hao *et al.*, 2011). In this study, driven by Illumina sequencing technology, DGE profiling created genome-wide expression profiles under B deficiency by sequencing ‘QY10’ and ‘W10’, which was indispensable for understanding the differential transcriptional response to B deficiency in these *B. napus* genotypes with contrasting B efficiencies.

B deficiency caused considerable differences in the transcript levels of a wide range of genes involved in several biological processes between ‘QY10’ and ‘W10’, which might arise from their differential sensitivities to B limitation conditions. Based on GO analyses of the DEGs, transporter, transferase, oxidoreductase, and kinase were the most enriched protein classes (Supplementary Fig. S5). Among them, particular attention was paid to the transporter and oxidoreductase categorizes. A total of 953 and 770 DEGs related to transporters were identified in the shoots and roots, respectively, of ‘QY10’ and ‘W10’ (Supplementary Fig. S4C). Given the crucial roles of roots in responses to B deficiency (Yang *et al.*, 2013), much greater attention was paid to the transporters in the root. Of special interest, more transporter genes, including potential B transporter genes (*BnaBOR1*s, *BnaNIP5;1*s, and *BnaNIP6;1*s) (Fig. 8E), with higher transcript levels were detected in ‘QY10’ (501) than ‘W10’ (269) (Supplementary Fig. S4B), which was assumed to be a key factor for the stronger resistance to B deficiency in the B-efficient genotype. In addition, ROS production in the roots was observed in response to nutrient deficiency, which may function as an important component in signalling nutrient deprivation (Schachtman and Shin, 2007). B deficiency triggered higher ROS accumulation in ‘W10’ relative to ‘QY10’ (Fig. 2A–D), showing the greater sensitivity of ‘W10’ to B deprivation, which might ultimately be attributable to its insufficient B uptake and movement by transporters. Meanwhile, more DEGs related to antioxidant enzymes with higher transcript levels were identified in ‘QY10’ than ‘W10’ (Supplementary Fig. S1) and the powerful ability of ‘QY10’ to scavenge ROS also highlighted its stronger tolerance to B deficiency.

### DGE-assisted QTL-seq analysis expedites the identification of candidate QTLs or genes underlying B efficiency in allotetraploid rapeseed

A combination of QTL analyses and transcriptome profiling has been used as a powerful approach for the rapid identification of functional polymorphisms and candidate genes for traits of interest (Jain *et al.*, 2014; Das *et al.*, 2016).

In this study, based on WGS of the B-efficient and B-inefficient DH line pools, QTL-seq revealed two prior candidate QTLs (*qBEC-C2a* and *qBEC-C2b*) with opposite additive effects for B efficiency on chromosome C2 (Fig. 9A). However, the genome complexity (A_n_A_n_C_n_C_n_, ~1,130Mb, 2*n*=4*x*=38) of allotetraploid rapeseed (Chalhoub *et al.*, 2014) causes great difficulty in the fine-mapping of QTLs for agronomical traits (Liu *et al.*, 2015), and the QTLs span extensive genomic regions (Fig. 9A) so that traditional QTL mapping could hardly delimit the QTL regions to several genes within a short time. Under these circumstances, we formulated DGE-assisted DEG analyses to expedite the identification of candidate genes for B efficiency; then, a nodulin 26-like intrinsic protein (NIP) gene (*BnaC2.NIP5;1*) and an ABC transporter gene (*BnaC2.ABCG21*) were identified as the prior candidates underlying *qBEC-C2a* and *qBEC-C2b*, respectively (Fig. 9E, H). In terms of the QTL *qBEC-C2a*, the candidate gene *BnaC2.NIP5;1* was highly homologous (92.8%) to *AtNIP5;1* (*AT4G10380*), which is an influx boric acid channel essential for efficient B uptake and normal plant development under B-limitation conditions in *Arabidopsis thaliana* (Takano *et al.*, 2006), and it has been assumed to function as an influx B transporter similar to AtNIP5;1. Although higher expression of *BnaC2.NIP5;1* was identified in the B-inefficient genotype ‘W10’ (Fig. 10B, C), it did not make ‘W10’ B-efficient (Fig. 1). On one hand, it may function as an intermediate signal triggered by B deficiency to induce the downstream genes (such as the genes for B transport and distribution), which further regulate B efficiency. On the other hand, B efficiency is controlled by multiple genes (Zhang *et al.*, 2014), which may include comprehensive roles of *BnaC2.NIP5;1* with other genes responsible for B uptake, transport, and distribution. The candidate *BnaC2.ABCG21* underlying the QTL *qBEC-C2a* was identified to be acting in a B-dependent manner (Fig. 10D, E) for the first time. Little has been known about the role of ABC (especially the G-subgroup) in B uptake and transport, although ABC transporter genes have been established to transport a wide range of compounds critical for successful adaptation to various terrestrial environments, including phytohormones, lignin precursors, secondary metabolites, hydrophobic compounds, and diverse phenolics, among others (Hwang *et al.*, 2016). Thus, much effort should be devoted to validating the roles of the nodulin 26-like intrinsic gene and the ABC transporter gene in B uptake and transport in *B. napus*. Additionally, the negative regulator genes for the candidates underlying the *qBEC-C2a* and *qBEC-C2b* QTLs remain to be functionally validated in future work.

Taken together, the strategy of QTL-seq-assisted identification of B-efficient candidate genes has shown great efficacy in the rapid genome-wide scanning of potential candidate gene(s) underlying trait-associated high-resolution robust QTL(s), thereby expediting the genomics-assisted breeding and genetic improvement of diverse plant species with complex genomes.

### A proposed model delineating the differential responses to B deficiency in rapeseed genotypes

**Fig. 11.**
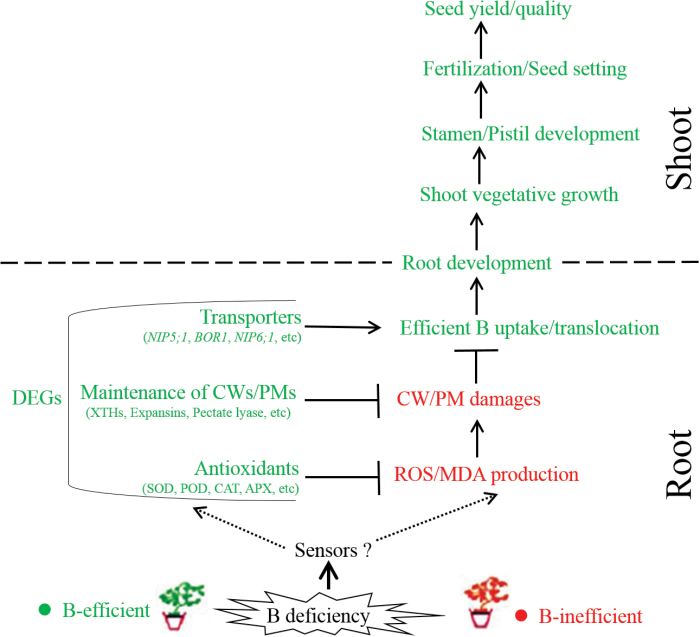
Proposed model delineating the differential responses to B deficiency in rapeseed genotypes. Green represents higher or more favourable levels in the B-efficient genotype, and red indicates higher levels in the B-inefficient genotype. ROS, reactive oxidative species; MDA, malondialdehyde; CW, cell wall; PM, plasma membrane; DEGs, differentially expressed genes; AGPs, arabinogalactan-proteins; POD, peroxidase; SOD, superoxide dismutase; APX, ascorbate oxidase; CAT, catalase. (This figure is available in colour at *JXB* online.)

It is universally acknowledged that nutrient perception, uptake, and transport are greatly dependent on roots in plant species (Sharp and Davies, 1979). A grafting experiment in a previous study demonstrated that B efficiency in *B. napus* was controlled primarily by the roots, which allowed greater B uptake and accumulation in B-efficient cultivars than B-inefficient cultivars under low-B conditions (Yang *et al.*, 2013). In our present study, the greater sensitivity of the B-inefficient genotype ‘W10’ to B deficiency resulted in elevated ROS and MDA accumulation in the roots (Fig. 2), which further caused abnormal cell morphologies, including disordered chloroplasts, shrunken PMs, and swollen CWs (Fig. 3). Furthermore, these greater damaging effects on the cell membrane integrity may pose a threat to B uptake and movement by transporters localized in the PMs (e.g. NIP5;1 and BOR1), which in turn would aggravate the harms caused by B deficiency. However, under the same conditions, the enhanced transcript levels of the genes related to antioxidant enzymes and the maintenance of CWs/PMs and B transporters (Fig. 8) conferred excellent tolerance against B deficiency in the B-efficient genotype ‘QY10’ (Figs 1–5), alleviating these detrimental effects of ROS/MDA production on the integrity of CWs/PMs and contributing to efficient B uptake and transport in the roots (Fig. 1I, J) and further facilitating the normal development of the vegetative and reproductive organs (Fig. 4). Taken together, we propose an integrated model to delineate the differential responses to B deficiency in rapeseed genotypes (Fig. 11), which also provides comprehensive insights into the pivotal roles of B in vegetative and reproductive development.

## Supplementary data

Supplementary data are available at *JXB* online.

Figure S1. Expression profiles of the differentially expressed genes related to antioxidant enzymes in the shoots and roots of the B-efficient genotype ‘QY10’ and the B-inefficient genotype ‘W10’.

Figure S2. Reproductive performance of the B-efficient genotype ‘QY10’ and the B-inefficient genotype ‘W10’ grown under a pot culture system.

Figure. S3. Annotations of insertions/deletions (InDels) and single nucleotide polymorphisms (SNPs) identified between the B-efficient genotype ‘QY10’ and the B-inefficient genotype ‘W10’.

Figure S4. Numbers of differentially expressed genes in the shoots and roots of the B-efficient genotype ‘QY10’ and the B-inefficient genotype ‘W10’.

Figure S5. Gene ontology (GO) enrichment analysis of differentially expressed genes in the shoots and roots between the B-efficient genotype ‘QY10’ and the B-inefficient genotype ‘W10’.

Figure S6. Frequency distribution of total dry weight in a 190-line DH population derived from the B-efficient genotype ‘QY10’ and the B-inefficient genotype ‘W10’.

Table S1. Primer sequences used for RT-qPCR assays in this research.

Table S2. Overview of reads generated from Illumina Hiseq 2500 for digital gene expression profiling.

Table S3. Annotated genes in the QTL *qBEC-C2a* region on chromosome C2.

Table S4. Annotated genes in the QTL *qBEC-C2b* region on chromosome C2.

Supplementary Data
