# Supplementary material for: Physiological, genomic and transcriptional diversity in responses to boron deficiency in rapeseed genotypes
Source: J Exp Bot. 2016 Sep 17;67(19):5769–84. doi: 10.1093/jxb/erw342 (PMC5066495; doi:10.1093/jxb/erw342)
Supplement: Supplementary Data [file supp_67_19_5769__index.html]

Physiological, genomic and transcriptional diversity in responses to boron deficiency in rapeseed genotypes — Physiological, genomic and transcriptional diversity in responses to boron deficiency in rapeseed genotypes — Supplementary Data 

# Physiological, genomic and transcriptional diversity in responses to boron deficiency in rapeseed genotypes

## Supplementary Data

Data files

- Supplementary\_Figures\_S1\_S6\_Tables\_S1\_S4.pdf - Supplementary Data
