# Supplementary material for: Physiological, genomic and transcriptional diversity in responses to boron deficiency in rapeseed genotypes
Source: J Exp Bot. 2016 Sep 17;67(19):5769–84. doi: 10.1093/jxb/erw342 (PMC5066495; doi:10.1093/jxb/erw342)
Supplement: Supplementary Data [file supp_erw342_Supplementary_Figures_S1_S6_Tables_S1_S4.pdf]

Supplementary data

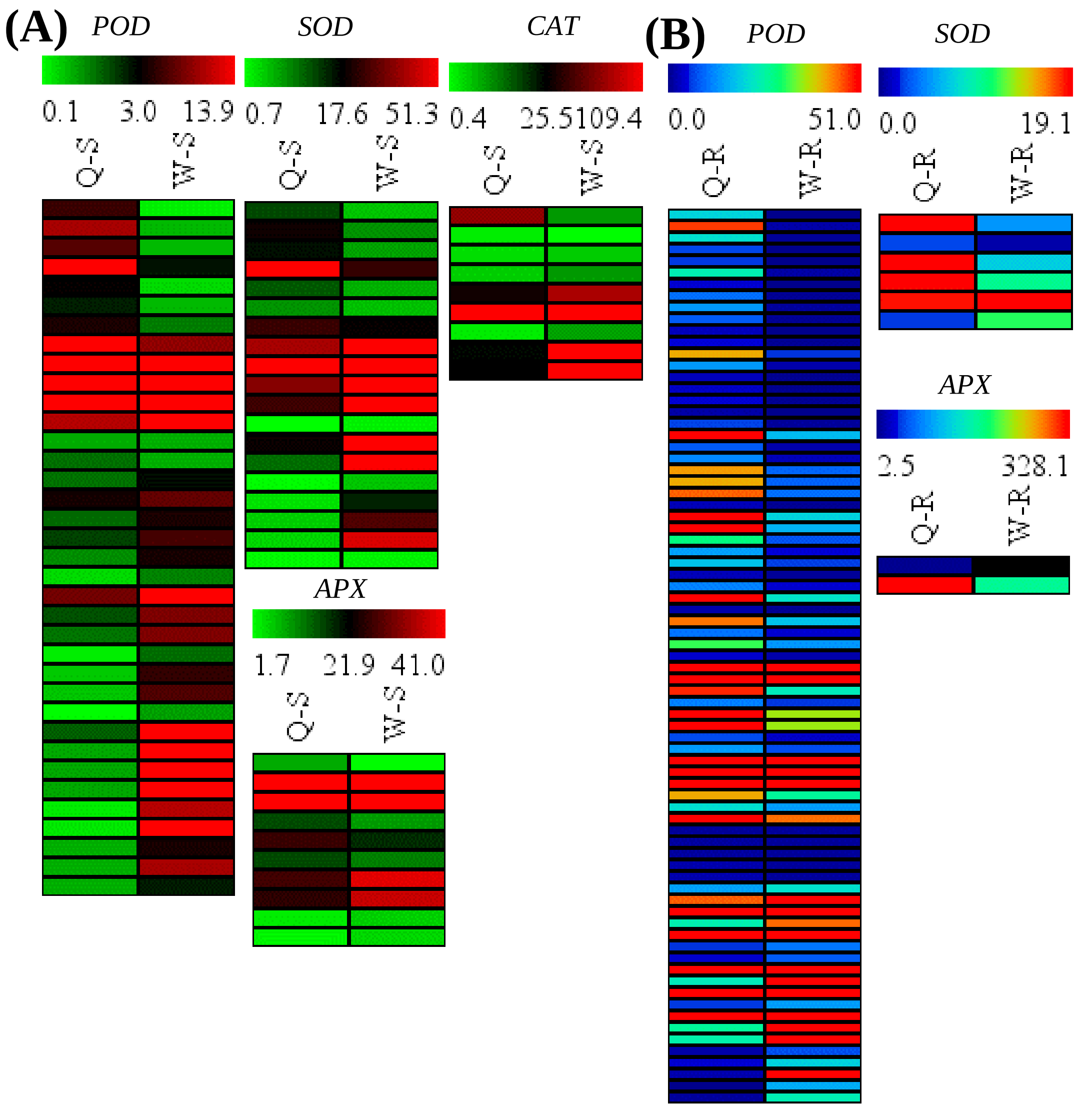

Supplementary Fig. S1 Expression profiles of differentially expressed genes (DEGs) related to antioxidant enzymes in the shoots (A) and roots (B) of the B-efficient genotype 'QY10' and the B-inefficient genotype 'W10', which were exposed to 10-day B deficiency (0.25 μM) condition. 'Q-S' and 'W-S' represent the DEGs in the shoots of 'QY10' and 'W10', and 'Q-R' and 'W-R' represent the DEGs in the roots of 'QY10' and 'W10'. POD: peroxidase; SOD: superoxide dismutase; APX: ascorbate oxidase; CAT: catalase.

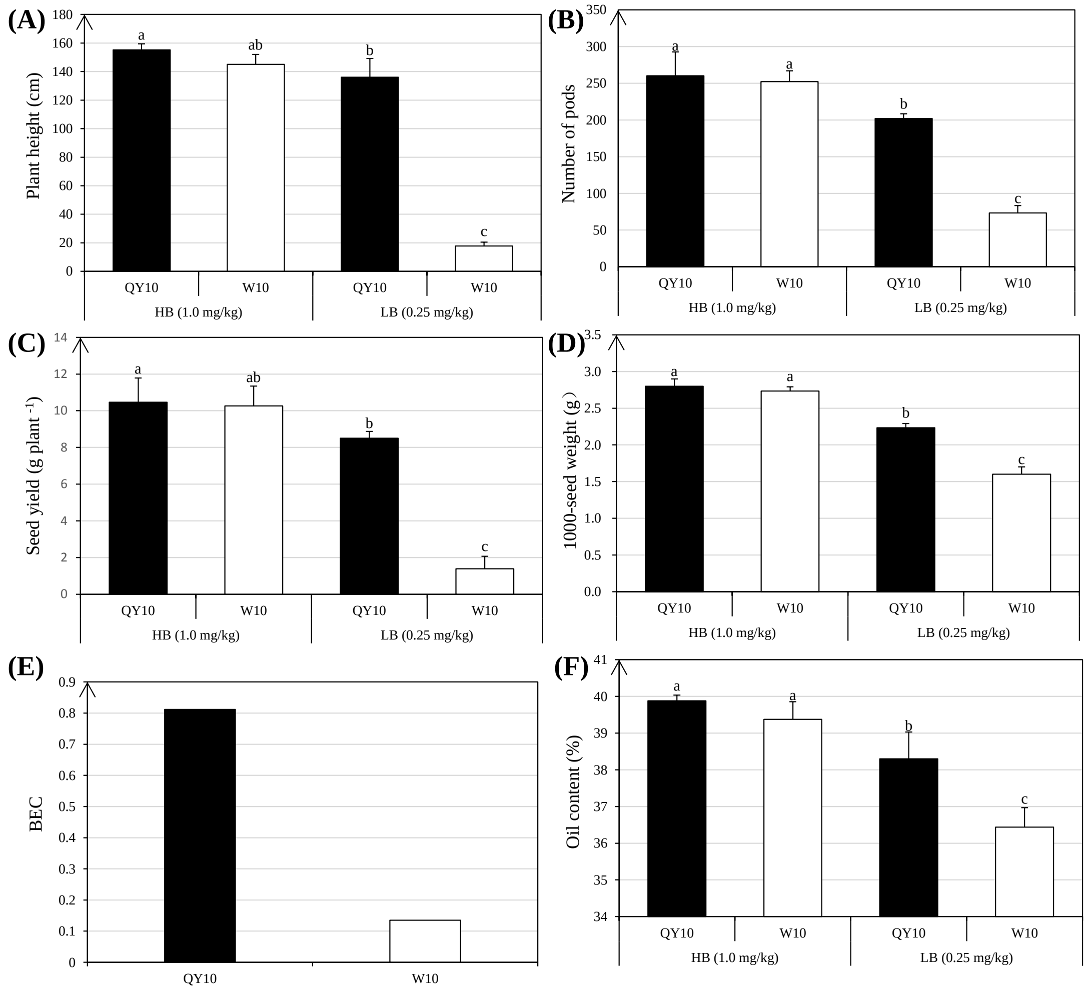

Supplementary Fig. S2 Reproductive performance of the B-efficient genotype 'QY10' and the B-inefficient genotype 'W10' grown under pot culture systems. (A) Plant height (cm); (B) number of pods; (C) seed yield; (D) 1000-seed weight; (E) BEC of 'QY10' and 'W10', B efficiency coefficient (BEC) = seed yield (0.25 mg B per kg soil) / seed yield (1.0 mg B per kg soil) (Hua *et al.*, 2016); (F) oil content. HB: high B, 1.0 mg B per kg soil; LB: low B, 0.25 mg B per kg soil. Bars denote the means (n = 3), and error bars denote the standard deviations (SD). The significance level was set at a *P*-value < 0.05.

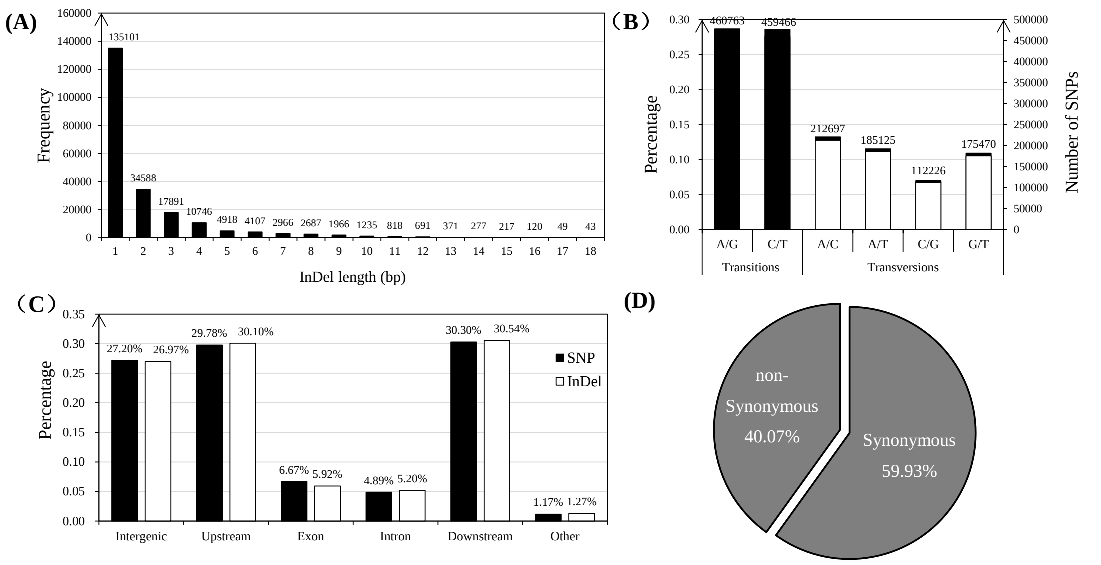

Supplementary Fig. S3 Annotations of insertions/deletions (InDels) and single nucleotide polymorphisms (SNPs) identified through the whole-genome re-sequencing (WGS) of the B-efficient genotype 'QY10' and the B-inefficient genotype 'W10'. (A) Frequencies and size (bp) of InDels; (B) frequencies of substitution types of SNPs. (C) Distribution of SNPs and InDels in different genomic regions. (D) The number of synonymous and non-synonymous SNPs detected within the coding sequencing (CDS) regions.

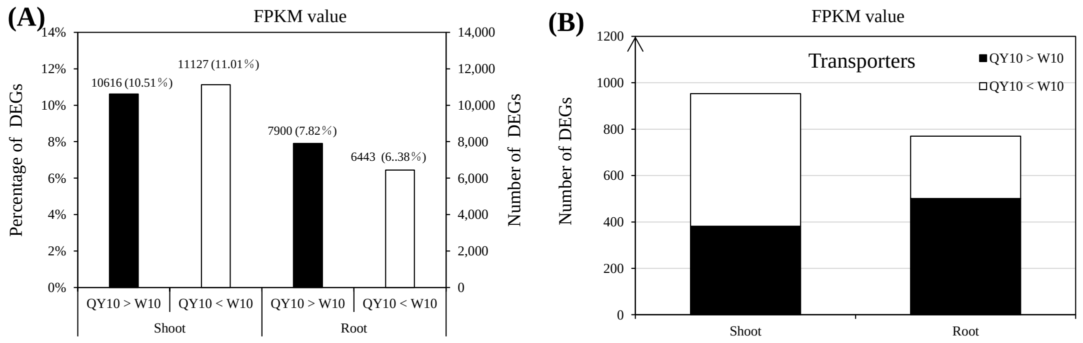

Supplementary Fig. S4 Numbers of differentially expressed genes (DEGs) in the shoots and roots of the B-efficient genotype ‘QY10’ and the B-inefficient genotype ‘W10’. (A) Number of total DEGs in the shoots and roots; (B) number of DEGs for transporters. “QY10 > W10” indicates that the transcript levels of the DEGs are higher in‘QY10’ than in ‘W10’, and vice versa.

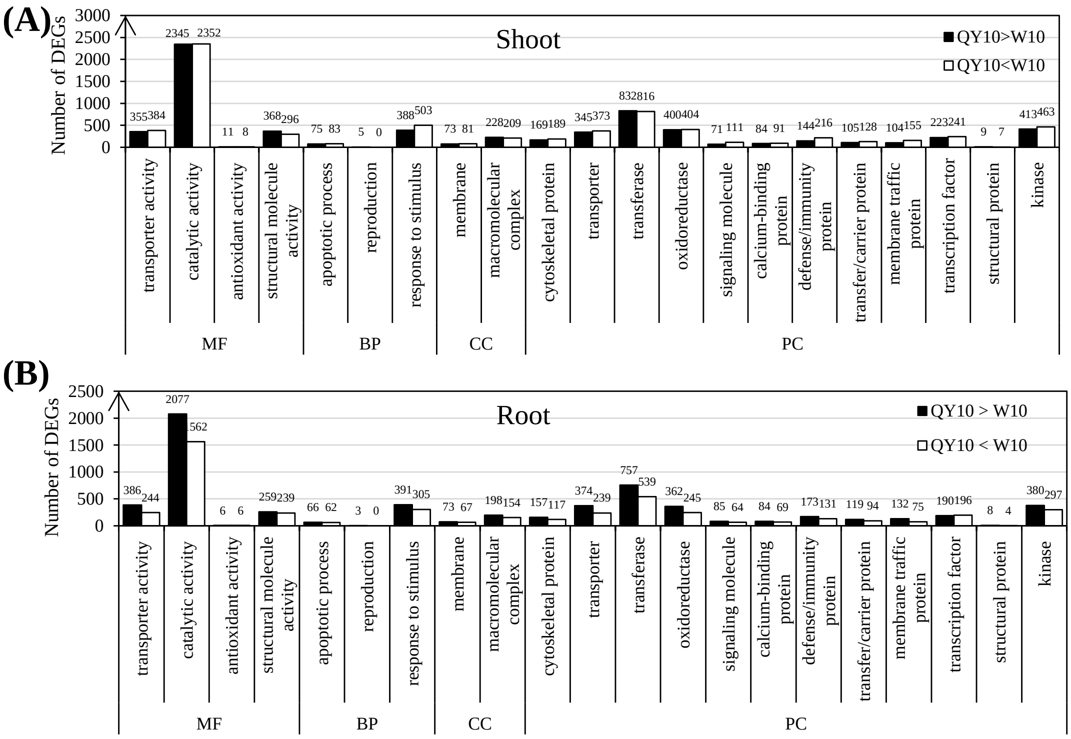

Supplementary Fig. S5 Gene ontology (GO) enrichment analysis of DEGs in the shoots (A) and roots (B) between the B-efficient genotype ‘QY10’ and the B-inefficient genotype ‘W10’. MF: molecular function; BP: biological process; CC: cellular component; PC: protein class. “QY10 > W10” indicates that the transcript levels of the DEGs are higher in ‘QY10’ than in ‘W10’, and vice versa.

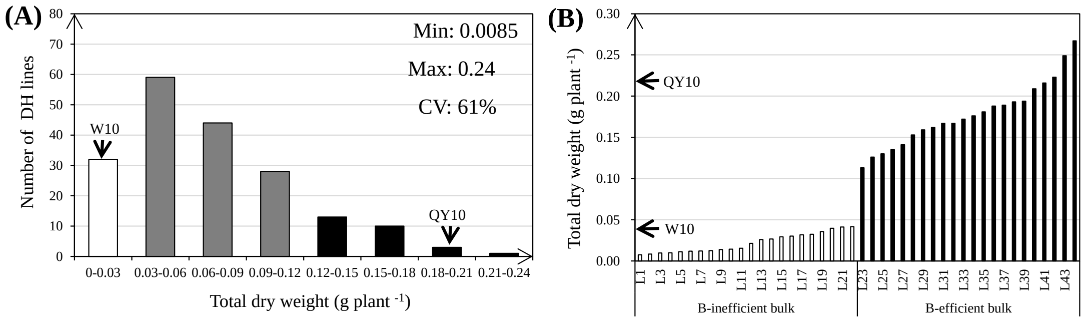

Supplementary Fig. S6 Frequency distribution of total dry weight in the 190-line DH population derived from the B-efficient genotype ‘QY10’ and the B-inefficient genotype ‘W10’. (A) Frequency distribution of total dry weight in the 190-line DH population, which were cultivated under hydroponic culture systems. The B-efficient and -inefficient pools (~30 lines for each pool) roughly selected from the 190-line DH population are denoted by black and white columns. (B) Validation of B efficiency of the B-efficient and –inefficient line pools through hydroponic culture. Total dry weights of ‘QY10’ and ‘W10’ are denoted by arrows.

Table S1 Primer sequences used for RT-qPCR assays in this research

Gene	Primer sequence (5'-3')	
	Forward	Reverse
Bnac2.NIP5;1	CCTAATTGGTAACGCGGCATGT	TCAGCGAAGGGTTTAAGTGAGCT
BnaC2.ABCG21	GACTTACAGTTGTAGTAACGATGTGTCA	GAATGCCACCATAAGAGGCCAG
Actin	ACAGTGTCTGGATCGGTGGTTC	TGCCTCATCATACTCAGCCTTG

Table S2 Overview of reads generated from Illumina Hiseq 2500 for digital gene expression (DGE) profiling

Sample name			Raw Reads	Clean reads	Clean bases	Error (%)	Q20 (%)	Q30 (%)	GC (%)
Shoot	QY10	R1	14,124,353	13,228,316	0.66 G	0.01	98.61	95.74	47.32
		R2	13,965,834	13,136,733	0.66 G	0.01	98.63	95.82	46.51
		R3	13,496,185	12,831,669	0.64 G	0.01	98.65	95.84	46.64
	W10	R1	13,637,492	12,998,934	0.65 G	0.01	98.65	95.8	47.54
		R2	14,233,990	13,461,872	0.67 G	0.01	98.65	95.83	46.31
		R3	13,156,784	13,049,106	0.65 G	0.01	98.71	95.86	47.39
Root	QY10	R1	15,085,715	15,051,356	0.75 G	0.01	98.57	95.74	45.41
		R2	13,181,542	13,141,067	0.66 G	0.01	98.63	95.76	46.32
		R3	13,380,622	13,337,441	0.67 G	0.01	98.64	95.78	46.35
	W10	R1	13,243,078	12,391,022	0.62 G	0.01	98.61	95.72	46.27
		R2	13,524,445	12,613,107	0.63 G	0.01	98.61	95.73	46.46
		R3	10,228,329	9,928,055	0.50 G	0.01	98.03	94.95	47.02

Table S3 Annotated genes in the QTL *qBEC-C2a* region on chromosome C2

Gene ID	Number of exons	CDS/aa size (bp/-)	*A. thaliana* homologs	E value	Putative protein function
BnaC02g26130D	7	1650/549	AT4G04540	0	Cysteine-rich kinase 39 (CRK39)
BnaC02g26380D	4	390/129	AT4G04780	2.0E-67	Mediator complex
BnaC02g27870D	2	204/67	AT1G48520	4.0E-10	Glutaminyl-tRNA synthase
BnaC02g28010D	3	354/117	-	-	Unknown
BnaC02g28790D	1	495/164	AT5G42820	6.0E-43	RNA binding
BnaC02g28840D	2	348/115	-	-	Unknown
BnaC02g28900D	2	744/247	-	-	Unknown
BnaC02g29210D	4	906/301	AT4G10380	1.0E-170	Nodulin 26-like intrinsic protein 5;1 (NIP5;1)
BnaC02g29860D	1	411/1136	AT5G42200	2.0E-78	RING/U-box superfamily protein

Table S4 Annotated genes in the QTL *qBEC-C2b* region on chromosome C2

Gene ID	Number of exons	CDS/aa size (bp/-)	*A. thaliana* homologs	E value	Putative protein function
BnaC02g32320D	2	276/91	AT2G34690	2.0E-14	ACCELERATED CELL DEATH 11 (ACD11)
BnaC02g32330D	3	381/126	AT2G34690	3.0E-46	ACCELERATED CELL DEATH 11 (ACD11)
BnaC02g33830D	6	1077/358	AT2G01260	4.0E-174	Unknown
BnaC02g33980D	3	543/180	AT2G01540	3.0E-119	Calcium-dependent lipid-binding protein
BnaC02g34120D	7	999/332	AT2G01880	0	Purple acid phosphatase 7 (PAP7)
BnaC02g34140D	8	1290/429	AT2G01900	0	Endonuclease/exonuclease/phosphatase
BnaC02g34260D	6	1482/493	AT2G02010	0	Glutamate decarboxylase 4 (GAD4)
BnaC02g34270D	3	258/85	AT5G16250	7.0E-34	Unknown
BnaC02g34720D	3	438/145	AT3G62050	7.0E-20	Glycosyl hydrolase
BnaC02g35410D	6	915/304	AT1G13580	2.0E-177	LAG1 longevity assurance homolog 3 (LAG13)
BnaC02g35560D	5	2058/685	AT3G25620	0	ATP-binding cassette (ABC) transporter
BnaC02g35830D	4	453/150	AT3G25930	2.0E-79	Alpha hydrolases-like protein
BnaC02g35980D	2	1311/436	AT3G26210	0	Cytochrome P450
